# Supersaturable organic-inorganic hybrid matrix based on well-ordered mesoporous silica to improve the bioavailability of water insoluble drugs

**DOI:** 10.1080/10717544.2020.1815898

**Published:** 2020-09-04

**Authors:** Qiaoli Wu, Disang Feng, Zhengwei Huang, Minglong Chen, Dan Yang, Xin Pan, Chao Lu, Guilan Quan, Chuanbin Wu

**Affiliations:** a College of Pharmacy, Jinan University, Guangzhou, China; b Department of Pharmacy, Zengcheng District People’s Hospital, Guangzhou, China; c School of Pharmaceutical Sciences, Sun Yat-sen University, Guangzhou, China

**Keywords:** Mesoporous silica, supersaturable, precipitation inhibitor, water insoluble drugs, bioavailability

## Abstract

Mesoporous silica with uniform 2-D hexagonal pores has been newly employed as facile reservoir to impove the dissolution rate of water insoluble drugs. However, rapid drug release from mesoporous silica is usually accompanied by the generation of supersaturated solution, which leads to the drug precipitation and compromised absorption. To address this issue, a supersaturated ternary hybrid system was constructed in this study by utilizing inorganic mesoporous silica and organic precipitation inhibitor. Vinylprrolidone-vinylacetate copolymer (PVP VA64) with similar solubility parameter to model drug fenofibrate (FNB) was expected to well inhibit the precipitation. Mesoporous silica Santa Barbara amorphous-15 (SBA-15) was synthesized in acidic media and hybrid matrix was produced by hot melt extrusion technique. The results of *in vitro* supersaturation dissolution test obviously revealed that the presence of PVP VA64 could effectively sustain a higher apparent concentration. PVP VA64 was suggested to simultaneously reduce the rate of nucleation and crystal growth and subsequently maintain a metastable supersaturated state. The absorption of FNB delivered by the organic-inorganic hybrid matrix was remarkably enhanced in beagle dogs, and its AUC value was 1.92-fold higher than that of FNB loaded mesoporous silica without PVP VA 64. In conclusion, the supersaturated organic-inorganic hybrid matrix can serve as a modular strategy to enhance the oral availability of water insoluble drugs.

## 1. Introduction

Oral administration is regarded as the most dominant and convenient drug delivery route, which is well accepted by patients and medical personnels (Lau et al., 2018). However, an increasing number of new pharmaceutical ingredients show very low water solubility, resulting in slow dissolution rate and poor oral bioavailability (Jennotte et al., 2020). Over the past decades, the manufacture of amorphous solid dispersions containing water insoluble drugs in appropriate materials has attracted much attention (Qi and Craig, 2016; Ma et al., 2020; Schittny et al., 2020). Besides the extensively explored organic polymeric materials, tremendous attention has been gained in adapting inorganic porous micro- and nano-particles for amorphous solid dispersion drug delivery (Biswas, 2017). The unique advantages of these materials, including porous interior, large surface area, and good biocompatibility, make them promising reservoir for encapsulating a large amount of therapeutic cargos (Dening et al., 2019; Cao et al., 2020).

Since the first report by Mobil corporation researchers in 1992 (Kresge et al., 1992), mesoporous silica has emerged as a novel modular inorganic platform owing to its widespread applications in biomedical field (Maleki et al., 2017). As compared with the traditional porous materials containing heterogeneous structure, mesoporous silica exhibits a highly ordered porous interior without interconnection among hundreds of nano-size channels (Chen et al., 2017), which can be adapted as storage host for various therapeutic drugs and simultaneously offer pathways for drug dissolution. When poorly soluble drug molecules were restricted into the inner space of mesoporous silica channels, intermolecular interaction resulting in crystallization could be effectively prevented (Mehmood et al., 2020). Therefore, the amorphous state could be maintained during the shelf-life and subsequently increase the drug dissolution rate after oral administration (Wang et al., 2012). In 2001, Vallet-Regi and colleagues first put forward the concept of utilizing mesoporous silica as drug delivery carrier (Vallet-Regi et al., 2001). Since then, the research on their biomedical applications has increased exponentially, and successfully applied to a variety of poorly water soluble drugs for improved dissolution (Abd-Elrahman et al., 2016; Bukara et al., 2016; Quan et al., 2016).

In a solid dispersion system, insoluble drug is usually dispersed at amorphous state in the solid carriers, and a supersaturated solution usually generate after fast dissolution (Dereymaker et al., 2017). Drug concentration in supersaturated solution is in excess of its crystalline drug solubility. In principle, high drug concentration exposed to the gastrointestinal (GI) tract leads to an increased oral adsorption (Strindberg et al., 2020). However, the supersaturated state is inherently thermodynamically unstable, which will gradually lead to precipitation of the water insoluble drugs prior to absorption, subsequently resulting in unsatisfactory bioavailability (Ilevbare et al., 2013; Feng et al., 2018). Therefore, the prolonged maintenance of supersaturated state in the GI tract would be beneficial for better absorption.

Nucleation and crystal growth are two stages involves in the drug precipitation (or crystallization) from supersaturated solution. At the beginning, the dissolved drug molecules aggregate into two- or three-dimensional small clusters (Xu and Dai, 2013), which continue to grow and subsequently form the nuclei. (Erdemir et al., 2009). At the second stage, the obtained nuclei will serve as centers of crystallization and attract growth units to arrange at the outer surface, eventually form a crystal structure (Lindfors et al., 2008). If the nucleation and/or crystal growth stages can be retarded or inhibited, the supersaturated state will be maintained for extended time period for better absorption. Recently, various pharmaceutical polymers have been confirmed to exhibit a substantial effect on inhibiting the precipitation even at very low concentrations. The polymers can occupy growth sites and subsequently act as physical barrier to prevent the accumulation of drug molecules to the surface of existing crystal lattice (Ilevbare et al., 2012). Several studies have clearly demonstrated that the amorphous solid dispersion formulated with polymeric precipitate inhibitor can result in better oral bioavailability (Kwon et al., 2019; Schver et al., 2020). Therefore, polymeric addictive could be integrated with mesoporous silica to build an organic-inorganic hybrid system for enhancing the oral absorption of water insoluble drugs.

Herein, the outstanding delivery capacity of mesoporous silica was combined with the precipitation inhibition property of vinylprrolidone-vinylacetate copolymer to build a supersaturated hybrid matrix. Fenofibrate (FNB) with poor solubility of 0.8 μg/mL (25 °C) was chosen as a model drug. Mesoporous silica was firstly synthesized and hybrid matrix was prepared by hot melt extrusion (HME) technique. *In vitro* dissolution profiles under sink and supersaturation condition were conducted to evaluate the hybrid system. The *in vivo* pharmacokinetic study was also conducted utilizing beagle dogs to confirm whether supersaturated system may have any advantages in enhancing oral absorption of FNB.

## 2. Materials and methods

### 2.1. Materials

Fenofibrate (FNB) was provided by Kaifeng Pharmaceutical Factory (Henan, China); Commercial product Lipanthyl^®^ capsule was obtained from Recipharm Fontaine (Chenove, France); Tetraethyl orthosilicate (TEOS) and triblock copolymer Pluronic^®^ P123 with average Mw = 5750 were purchased from Sigma-Aldrich (St Louis, MO); Kollidon VA64 (Vinylprrolidone-vinylacetate copolymer, PVP VA64) was kindly donated by BASF (Ludwigshafen, Germany). All other materials used in this study were of reagent grade and utilized without any purification process.

### 2.2. Synthesis and characterization of SBA-15

Santa Barbara Amorphous-15 (SBA-15) was synthesized utilizing TEOS as silicon source and P123 as template according to the previous publication with minor modifications (Zhao et al., 1998). In brief, a defined amount of P123 (8.0 g) was added into 365 mL of hybrochloric acid solution (2 M) at 40 °C in a three-necked round bottom flask with constant stirring until a clear light blue solution was formed. Then, 17.53 g of TEOS was added dropwise, followed by stirring for 24 h at 40 °C. Subsequently, the obtained mixture was kept crystallization with gentle stirring for 2 days at 100 °C. After centrifugation, the white product was washed by deionized water for several times, rinsed with ethanol, and dried at 60 °C. Finally, the collected as-synthesized SBA-15 silica particles were allowed to calcine at 550 °C for at least 10 h to completely eliminate the structure-directing template P123.

The macroscopic morphology feature of samples was characterized by scanning electron microscopy (SEM, JSM-6330F, Japan). All samples were deposited on a brass stub and sputter-coated with gold film before imaging. The porous structure of the particles was observed by transmission electron microscopy (TEM, Tecnai G2 TF20, Japan) with an accelerated voltage of 200 kV. The mesostructure ordering was also measured using a small-angle X-ray diffractometer (XRD, D/MAX 2200VPC, Japan) utilizing Cu Kα radiation (40 Kv and 10 mA) at a scanning rate of 0.5°/min. The detailed information of the porous interior was measured by nitrogen absorption-desorption isotherms using a surface area and porosimetry analyzer (ASAP 2460, USA), with the pore volume, surface area, and pore size distribution calculated according to the isotherms.

### 2.3. Drug loading to SBA-15

Water insoluble drug FNB was loaded into the interior channels of SBA-15 employing the widely used wetness impregnation method. In brief, SBA-15 powder was added into the FNB ethanol solution at a concentration 5 mg/mL, followed by continuous magnetic stirring at ambient temperature for 24 h. Then, the volatile ethanol was completely removed at 40 °C on a rotary evaporator (IKA RV10 digital, Germany).

### 2.4. Preparation of organic-inorganic hybrid matrix

The ternary hybrid matrix (THM) was prepared by HME method using a conical twin-screw hot melt extruler (Thermo Electron GmbH, Karlsruhe). The mixture of FNB loaded SBA-15 and PVP VA64 were manually prepared and fed into the hopper, then extruded at 130 °C with an optimal screw rate of 80 rpm. The melt extrudates were collected by aluminum plate, allowed to cool at ambient temperature, then pulverized and passed through a 100-mesh sized sieve to obtain a powder sample for further evaluation. The composition of different hybrid matrix prepared by HME was shown in Table 1.

**Table 1.** The composition and powder properties of different formulations (Mean ± S.D., *n* = 3).

Formulation		Angle of repose (°)	Contact angle (°)
Code	FNB:SBA-15:PVP VA 64 (w: w: w)		
FNB	1: 0: 0	–	87.2 ± 0.4
F1	2: 1: 7	37.12 ± 0.37	29.9 ± 0.1
F2	2: 2: 6	35.21 ± 0.48	36.6 ± 1.6
F3	2: 3: 5	32.53 ± 0.51	39.6 ± 0.5
F4	2: 4: 4	31.65 ± 0.21	42.9 ± 0.8
FNB-SBA-15	2: 8: 0	45.79 ± 1.28	–

### 2.5. Solid state characterization of samples

#### 2.5.1. Macroscopic morphology feature

The macroscopic morphology feature of raw FNB, FNB loaded SBA-15, and hybrid matrix was evaluated by SEM. All samples were deposited on the brass stub surface, followed by sputter-coating with a thin gold film for two cycles.

#### 2.5.2. Physical state evaluation

The crystalline properties of FNB powder, FNB loaded SBA-15, and hybrid matrix were analyzed by XRD (D/MAX 2200VPC, Japan) with Cu Kα radiation. The diffraction patterns of samples were examined from 5° to 40°, and the scanning rate was set as 5°/min.

The thermal properties of the samples were analyzed using differential scanning calorimetry (DSC, NETZSCH STA409, Germany). Accurately weighed samples (approximately 5 mg) were deposited to the special aluminum pans followed by gradually heating to 120 °C at a rate of 10 °C/min with nitrogen atmosphere of 40 mL/min.

### 2.6. Physical properties of powders

Powdered materials of different formulations were measured for angle of repose applying a BT 1000 powder analyzer (DanDong Better size Instruments, China). The wettability of the powders was evaluated using a contact angle analyzer (Dataphysics OCA-35, Filderstadt, Germany). Prior to measurement, 300 mg of powder was dried and then compressed into a slice with a diameter size of approximately 12 mm under a pressure of 10 MPa for one minute. A small drop of pure water (1 μL) was instilled onto the slice surface. The dynamic wetting angle was recorded using a video system at the speed of 6 frames/min.

### 2.7. Dissolution test under sink condition

The dissolution test under sink condition was conducted utilizing a ZRS-8G dissolution tester equipped with USP paddle apparatus (TDTF, Tianjin, China) at 37 °C and 100 rpm. The samples containing equivalent amounts of FNB (20 mg) were added in 900 mL of release medium consisted of deionized water with sodium dodecyl sulfate (0.3%, w/v). Aliquot release medium (5 mL) was withdrawn at predetermined time intervals and an equal volume of fresh medium was added to compensate the sampling loss. The obtained samples were filtered through a 0.22 μm microporous membrane, and determined at 288 nm by UV spectrophotometry (TU-1901, Beijing, China).

### 2.8. Dissolution test under supersaturation condition

Supersaturation dissolution test was also carried out utilizing a USPII paddle method (37 °C ± 0.5 °C, 100 rpm, 900 mL of deionized water). A defined amount of sample equivalent to 30-times the solubility of FNB was added into the dissolution vessel. Aliquot release medium (5 mL) was withdrawn at predetermined time intervals. The obtained samples were immediately filtered through a 0.22 μm Millipore membrane, and discarded the primary filtrate. To prevent precipitation, a define volume of absolute ethanol was immediately added to 0.5 mL of filtrate and the FNB concentration was analyzed using HPLC method.

### 2.9. *In vivo* pharmacokinetic study

#### 2.9.1. Administration schedule

All the animal experimental procedures utilized in this study were approved by the Institutional Animal Care and Use Committee of Sun Yat-sen University. For the *in vivo* bioavailability study, six healthy beagle dogs (about 10-12 months of age) weighing 10 ± 0.5 kg were employed in the randomized crossover design with a two-week washout period. The dogs were allowed to fast for 12 h before the experiment but with water provided. A single oral dose of hard capsules containing hybrid matrix, FNB loaded SBA-15, and commercial FNB capsules containing 100 mg of FNB was administrated along with water (approximately 20 mL). Food was returned at 4 h post-dosing. At 0, 0.25, 0.50, 0.75, 1, 1.5, 2, 3, 4, 6, 8, and 24 h after administration, 5 mL of blood samples were taken out from the cephalic vein of the hind leg. The plasma samples were collected by centrifugation at 5000 rpm for 5 min and subsequently kept at −20 °C for further quantitative analysis.

#### 2.9.2. Measurement of plasma FNB concentration

The HPLC system was equipped with LC-20AT pump, SPD-20A ultraviolet-visible detector, DGU-20A5R system controller, and symmetry^®^ C18 column (4.6 × 250 mm, 5 μm). The UV-detection was conducted at 286 nm using a mobile phase containing acetonitrile and 0.1% acetic acid (65: 35, v/v). The working temperature of column was set as 40 °C and the record time was 30 min.

To analyze the drug content in plasma, 500 μL of sample was mixed with 50 μL of prepared methanol solution containing indometacin (50 μg/mL) as internal standard, followed by addition of hybrochloric acid solution (200 μL, 1 M). The obtained mixture was allowed to vortex for 1 min, followed by adding extraction solvent diethyl ether (4 mL) and kept vortexed for another 5 min. The organic layer was collected by centrifugation for 5 min at 8000 rpm and further evaporated under nitrogen atmosphere at 40 °C. Two hundred microliter of mobile phase was added to dissolve the obtained residue by under continuous vortex. After centrifugation at 15000 rpm for another 5 min, the supernatant was used for HPLC detection.

### 2.10. Statistical analysis

The pharmacokinetic parameters were calculated by the non-compartmental model utilizing WinNonlin V. 3.3 software (Pharsight, Sunnyvale, California). The *C*_max_ and *T*_max_ were directly acquired from the plasma concentration versus time profile. While the AUC value was calculated from time 0 to infinity by linear trapezoidal rule.

Data were expressed as mean ± standard derivation (SD). The statistical analysis was perfomed by a one-way ANOVA test using least significant difference post-test following normality and equal variance tests (SPSS 13.0). *P* values of <0.05 were considered to indicate statistically significant differences.

## 3. Results and discussion

### 3.1. Synthesis and characterization of SBA-15

**Figure 1.**
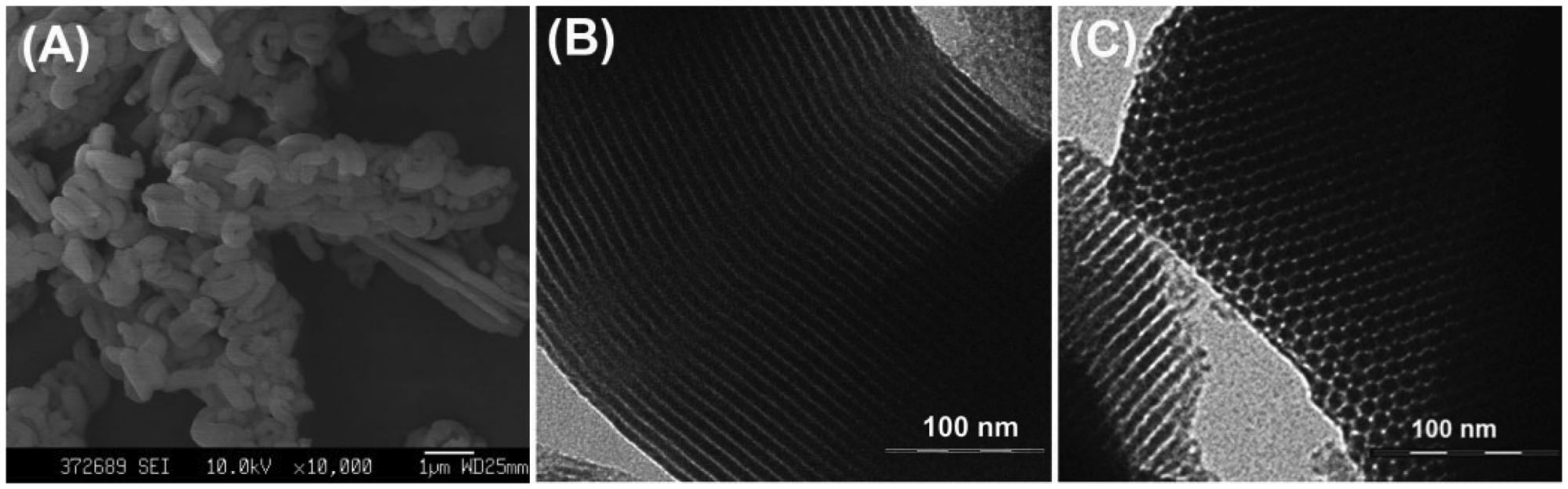
The macroscopic characterization of SBA-15. (A) SEM image of SBA-15; TEM images of SBA-15 shown at (B) parallel and (C) vertical orientation, respectively.

**Figure 2.**
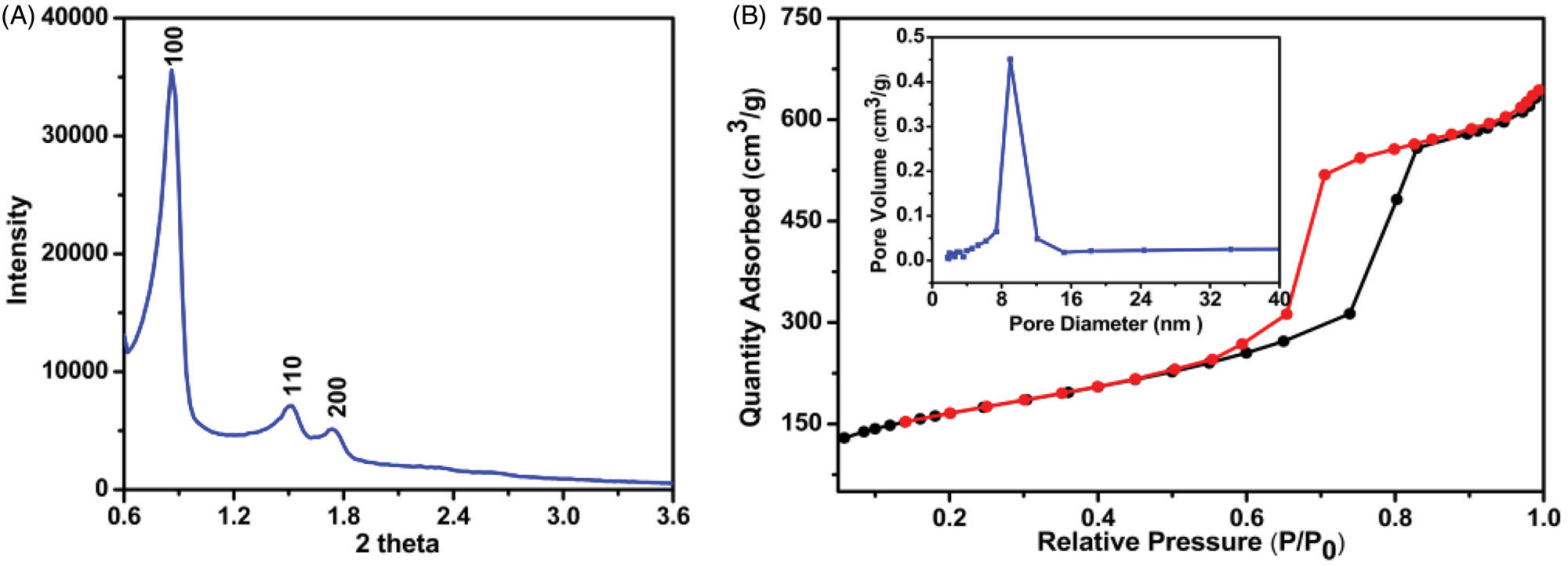
The structure characterization of SBA-15. (A) Small-angle XRD result of SBA-15; (B) Nitrogen adsorption (■)-desorption (●) isotherms of SBA-15, and the insert shows the pore size distribution.

In this study, SBA-15 with mesoporous structure was derived from TEOS and supermolecular assemblies of block-copolymer P123, which served as the silica precursor and structure-directing template, respectively. After forming pre-synthesized particles consisted of organic P123 micelles occupied by inorganic silica walls, the template was eliminated at 550 °C to expose the porous channels. As revealed in SEM image (Figure 1(A)), SBA-15 appeared as rod-shaped particles with length of approximately 0.5-1.5 µm. TEM image was applied to investigate the interior porous structure. The highly ordered hexagonal lattice and straight long-range fringes at vertical and parallel orientation were clearly observed (Figure 1(B,C)), which confirmed the existence of 2-dimensional hexagonal mesopores (Yu and Zhai, 2009). The small-angle XRD results of SBA-15 (Figure 2(A)) showed three well resolved peaks indexed as (100), (110), and (200) diffractions of *p*6mm hexagonal symmetry, suggesting a highly ordered porous structure (Moritz and Łaniecki, 2012). N_2_ adsorption-desorption measurement was performed to obtain the quantitative information about the mesopores. As revealed in Figure 2(B), SBA-15 showed the typical type-IV isotherms with an obvious H1 hysteresis loops at a relative pressure in the range of 0.55–0.85, indicating the porous interior of SBA-15 with regular pore size distribution. The mean pore size, pore volume, and surface area of SBA-15 were calculated as 9.00 nm, 0.45 cm^3^/g, and 680.55 m^2^/g, respectively. These results indicated that SBA-15 with highly ordered porous interior and large surface area can serve as versatile carrier to constrain drug molecules.

### 3.2. Optimization of organic-inorganic hybrid matrix

PVP VA64, a hydrophilic vinylprrolidone-vinylacetate copolymer extensively used as carrier for amorphous solid dispersion, was employed in this study as the precipitation inhibitor. The supersaturable organic-inorganic hybrid matrix was prepared by HME according to the composition displayed in Table 1. For all the formulations obtained, 98%∼102% of the theoretical drug amount were recovered after processing at 130 °C. 20% FNB-loaded SBA-15 without polymer (FNB-SBA-15) prepared by wetness impregnation method was used as the control group.

Powder characteristics were also investigated to estimate the practicality of different formulations. The angle of repose for FNB-SBA-15 was 45.79 degrees (Table 1), confirming poor flowability of the system. The smaller angle of repose represents better flowability, and it is generally accepted that an angle of repose smaller than 40 degrees can meet the needs of pharmaceutical industry production process. After preparation into hybrid solid dispersion, the angle of repose for the obtained sample reduced to less than 37.12 ± 0.37 degrees, and further increasing the percentage of SBA-15 led to smaller angle of repose.

Powder wetting is an important prerequisite for the release of any solid state drug powder. Therefore, water contact angle was analyzed in this study to investigate the wettability of the powders. A large contact angle typically suggests the poor wettability of the system. As shown in Table 1, the contact angle of raw FNB was as large as 87.2 ± 0.4 degrees, confirming its hydrophobic nature. The contact angle of FNB-SBA-15 was not recorded due to the unsuccessful pellet formation during the compression process. Formulating into ternary hybrid matrix significantly decreased the water contract angle of FNB formulations which is in the sequence of F1 < F2 < F3 < F4, demonstrating the enhanced wettability. In consideration of both the flowability and wettability of hybrid matrix, F3 was selected as the optimal formula for further analysis.

### 3.3. Solid state characterization of samples

#### 3.3.1. Macroscopic morphology feature

**Figure 3.**
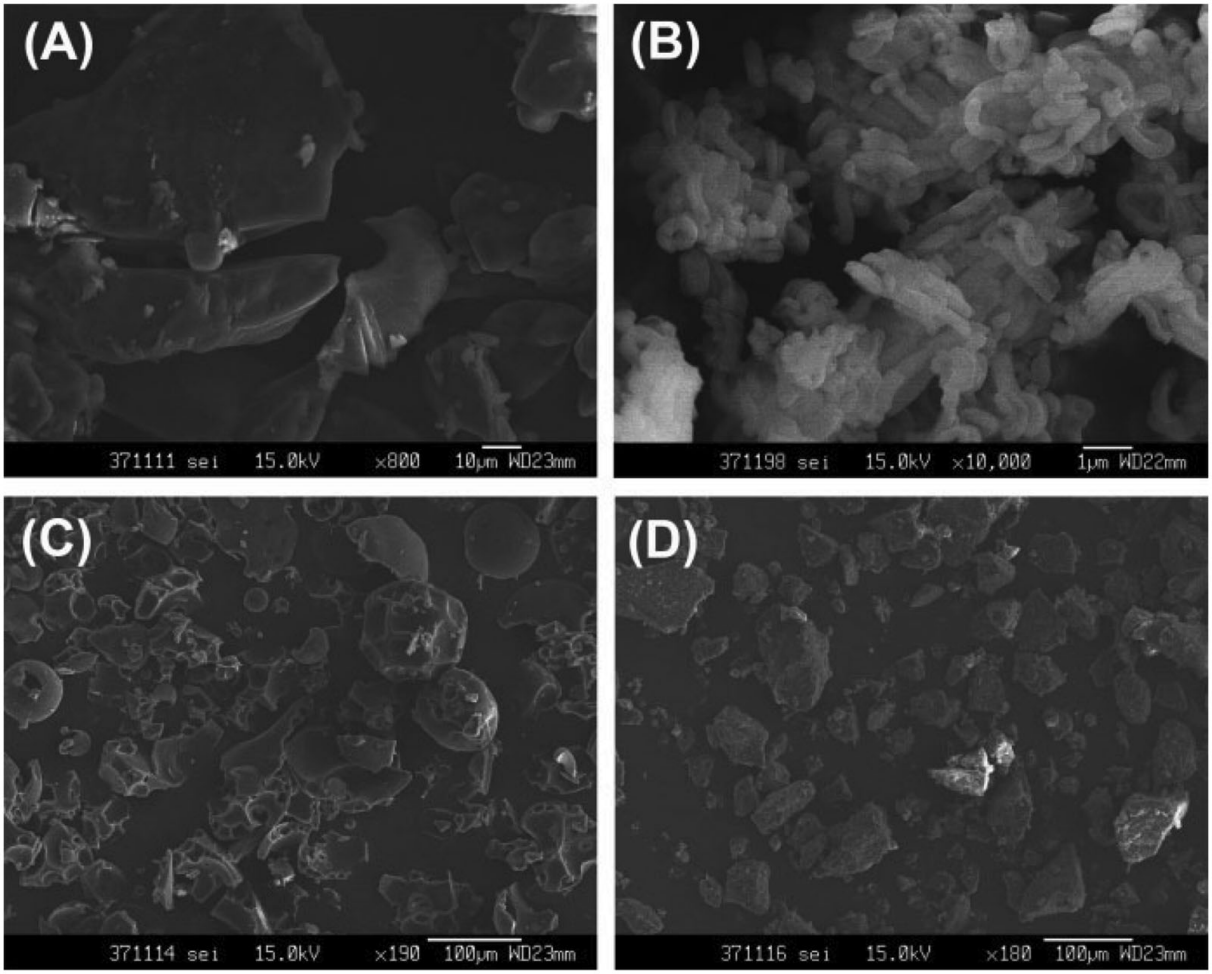
SEM images of FNB (A), FNB loaded SBA-15 (B), PVP VA 64 (C), and ternary hybrid matrix (D).

The SEM images of raw FNB, PVP VA64, FNB-SBA-15, and hybrid matrix were shown in Figure 3. The crystalline FNB appeared as well-defined prism block with smooth surfaces. PVP VA64 appeared as irregular spherical morphology accompanied with fragments, and FNB-SBA-15 possessed rod-like particles without any crystalline appearance. The morphological characteristics of FNB and carriers disappeared after feeding into hot melt extruder to form ternary hybrid matrix, indicating the high disperse ability of FNB into the interior channels of SBA-15 and the polymer matrix of PVP VA64.

#### 3.3.2 X-ray powder diffraction (XRD)

**Figure 4.**
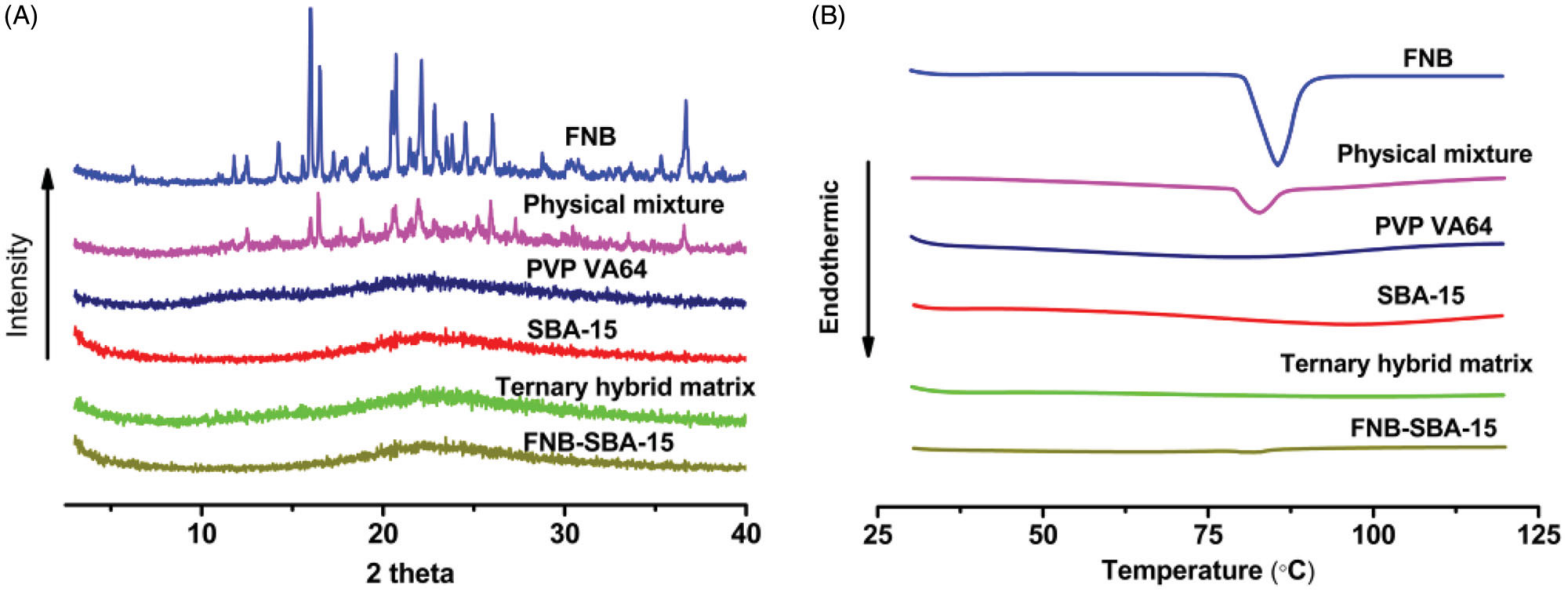
The physical state characterization. (A) Wide-angle XRD results of FNB, SBA-15, PVP VA64, FNB loaded SBA-15, physical mixture, and ternary hybrid matrix; (B) DSC curves of SBA-15, PVP VA64, FNB, FNB loaded SBA-15, physical mixture, and ternary hybrid matrix.

XRD patterns were conducted to investigate the existence of FNB crystalline phase. As shown in Figure 4(A), both SBA-15 and PVP VA64 did not exhibit any diffraction peaks because of the amorphous nature. By contrast, sharp distinct peaks corresponding to the FNB crystals were clearly shown in both raw FNB powder and the physical mixture. And no obvious diffraction peaks were detected for FNB-SBA-15 and hybrid solid dispersion, indicating the complete conversion of raw FNB from crystalline to molecular or amorphous state.

#### 3.3.3. Differential scanning calorimetry (DSC)

The physical state of FNB in the formulations was further evaluated using DSC and the result was presented in Figure 4(B). The crystalline nature of pure FNB can be easily distinguished by a sharp endothermic peak at 85.5 °C. An endothermic peak at same position was also observed in the physical mixture due to the existence of crystalline FNB, although the peak intensity was significantly decreased due to the dilution effect. In contrast, no endothermic peak was detected in the thermogram for FNB-SBA-15 and solid matrix, confirming the absence of any crystalline phase of FNB. Both the XRD and DSC results indicated that FNB in the hybrid solid dispersion was completely transformed into a molecular or amorphous state.

### 3.4. Dissolution test under sink condition

**Figure 5.**
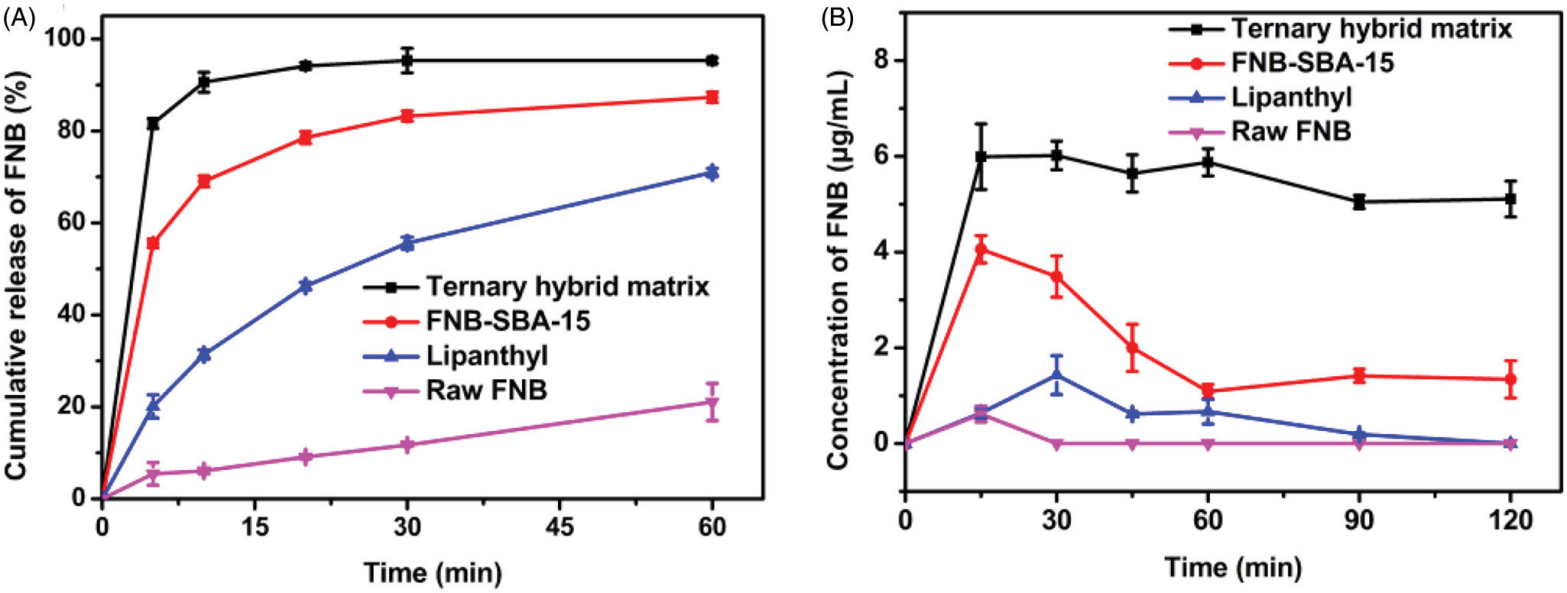
Dissolution test under different conditions. (A) Dissolution profiles of FNB from various samples under sink condition; (B) Apparent drug concentration versus time from *in vitro* supersaturation dissolution test (mean ± S.D., *n* = 3).

The *in vitro* dissolution profiles of different samples were investigated under sink condition in deionized water with addition of 0.3% (w/v) SDS. After 5 min, drug release from hybrid matrix and FNB-SBA-15 reached 81.61% and 55.58%, respectively. The drug release from Lipanthyl^®^ and crystalline FNB powder was recorded as 20.10% and 5.42%, respectively (Figure 5(A)). FNB, which belongs to BCSII class, has very poor water solubility (0.8 μg/mL, 25 °C), and resulting in the slowest dissolution rate under same condition as expected. And the relative enhancement of the dissolution rate from Lipanthyl^®^ is related to its pronounced particle size reduction through micronization. Compared to crystalline raw FNB and the commercial product, the FNB loaded SBA-15 showed a remarkably faster drug release rate. It spent 1 h for the commercial product to release 70% FNB, while only 10 min was required for the FNB-loaded SBA-15. This is mainly ascribed to the conversion of crystalline FNB to its amorphous or molecular counterpart, thus increased the solubility and dissolution rate. Additionally, the large surface area of SBA-15 containing abundant silanol group also contributed to the wettability improvement of the hydrophobic drug. Although the pasted clumping was formed in hybrid system, which was reported to prevent the permeation of dissolution medium and delay the dissolution rate (He et al., 2010). The influence of the pasted clumping was negligible here because it was dissolved within 5 min. Therefore, in addition to the drug state conversion, the fast drug release from hybrid matrix was ascribed to the combined effect of the unique porous structure of SBA-15 and the hydrophilic nature of PVP VA64.

### 3.5. Dissolution test under supersaturation condition

Since the supersaturated solution generated by fast dissolution is thermodynamically unstable, the drug molecules have a tendency to precipitate prior to absorption. Therefore, for better absorption, the ideal formulations should maintain the supersaturated state in the GI tract for long enough time. To evaluate the duration of the supersaturated state, the dissolution study under supersaturation condition was conducted to obtain the apparent drug concentration-time profiles. As shown in Figure 5(B), both crystalline FNB and the commercial product displayed very low level of dissolution throughout the whole period of 2 h. The apparent FNB concentration of FNB-SBA-15 was initially about 4.06 μg/mL at 10 min, and decreased rapidly to 1.34 μg/mL at 2 h. By contrast, the hybrid matrix showed a consistently higher apparent drug concentration. In particular, the concentration of FNB was 6.00 μg/mL at 10 min, and gradually decreased to 5.11 μg/mL at 2 h. These results clearly indicated that the presence of PVP VA64 can retard the FNB precipitation process and subsequently maintain a higher apparent concentration over approximately 2 h.

### 3.6. Mechanism of precipitation inhibition

Maintaining supersaturated state by addition of appropriate polymers depends on their capacity to retard the nucleation or crystal growth. After quantifying the impact of a series of polymeric additives on the nucleation behavior of different compounds, Taylor *et al* (Ilevbare et al., 2013) found that the polymer with hydrophobicity similar to the solute could maximize the polymer-solute interaction and subsequently inhibit the recognition and accumulation of drug molecules into small nuclei. The solubility parameter (SP) is commonly employed to evaluate the relative hydrophobicity of polymers and drug molecules. In general, a compound with lower SP value is proved to be more hydrophobic. Specifically, the SP value of water is reported to be 49.01 (mJ^1/2^⋅m^−3/2^). In this study, the SP value of polymer PVP VA64 and model drug FNB were calculated by Van Krevelen and Hoftyzer’s group contribution method, which was expressed by the following equation (Adamska and Voelkel, 2005):

$$\delta^{2}=\delta_{d\;}^{2}+\delta_{p}^{2}+\delta_{h}^{2}$$

where,

$$\delta_{d}=\frac{\sum F_{\mathrm{di}}}{V}\;\delta_{p}=\frac{\sqrt{\sum F_{\mathrm{pi}}^{2}}}{V}\;\delta_{h}=\sqrt{\frac{\sum E_{\mathrm{hi}}}{V}}$$

In this equation, δ represents the total solubility parameter, $\delta_{d},$ $\delta_{p},$ and $\delta_{h}$ represent the contribution from dispersion forces, polar forces, and hydrogen bonding, respectively. $F_{\mathrm{di}}$ and $F_{\mathrm{pi}}$ represent molar attraction constant due to dispersion component and polar component, respectively. $E_{\mathrm{hi}}$ represents hydrogen bonding energy, and V represents the molar volume. As shown in Table 2, the SP value of PVP VA64 is 19.04 (mJ^1/2^⋅m^−3/2^)^,^ which is quite comparable to that of FNB (the Δδ was only 0.06 (mJ^1/2^⋅m^−3/2^). Therefore, PVP VA 64 could mix with solute molecules effectively and subsequently hinder the reorganization of pre-nucleation aggregates into small nuclei, which is usually regarded as the rate-limiting stage of precipitation.

**Table 2.** Van Krevelen and Hoftyzer’s solubility parameter (δ, mJ^1/2^⋅m ^− 3/2^) of FNB and PVP VA 64.

	*δ_d_*	*δ_p_*	*δ_h_*	*δ*	Δδ
FNB	17.89	4.11	4.85	18.98	0.06
PVP VA 64	15.82	0.67	10.58	19.04	

**Figure 6.**
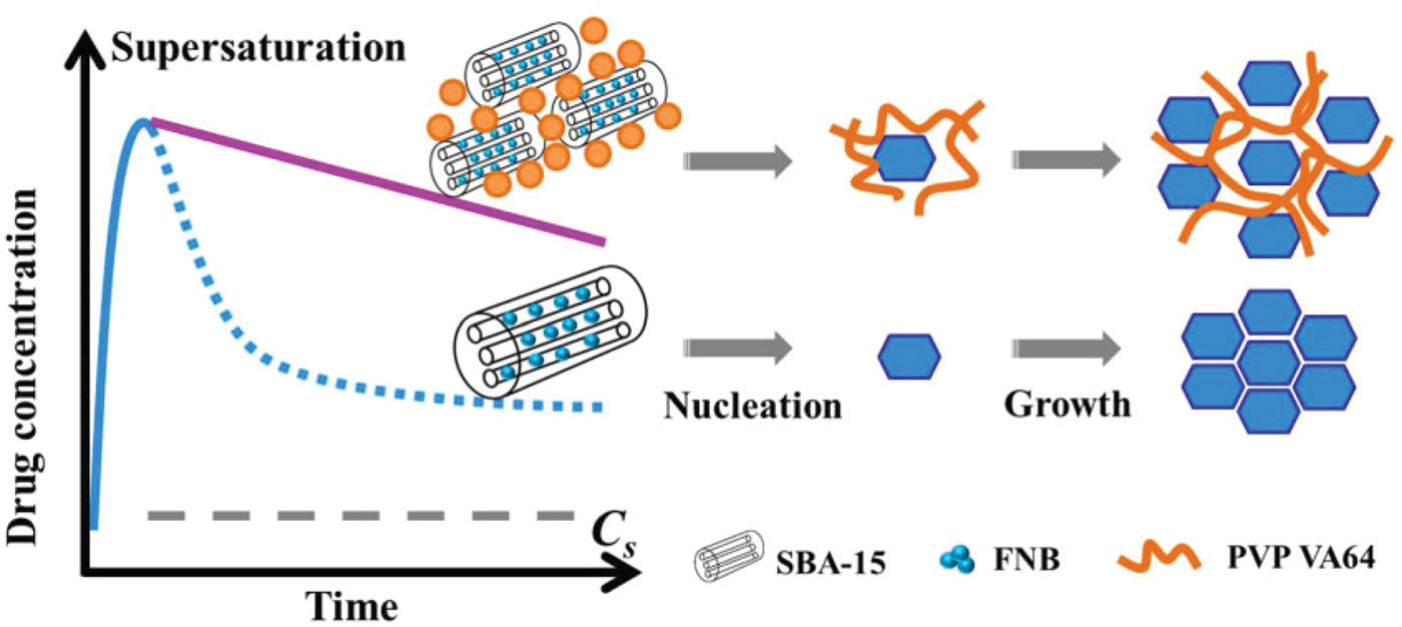
Schematic showing the mechanism of precipitation inhibition.

There is a general consensus that polymeric addictive should have an optimal hydrophobicity level to be served as an ideal inhibitor for crystal growth (Ilevbare et al., 2012). Polymer with higher hydrophilicity will tend to interact more favorably with water molecules. Whereas polymer with higher hydrophobicity is prone to exhibits a relatively higher affinity for other polymer units. PVP VA 64 with moderate hydrophobicity can retard the accumulation of drug molecules onto the crystal lattice by absorption to the crystal surface and thus serving as a mechanical barrier. These results indicated that PVP VA 64 was able to simultaneously reduce the rate of nucleation and crystal growth, and consequently leading to an efficient inhibition effect (Figure 6).

### 3.7. *In vivo* pharmacokinetic study

**Figure 7.**
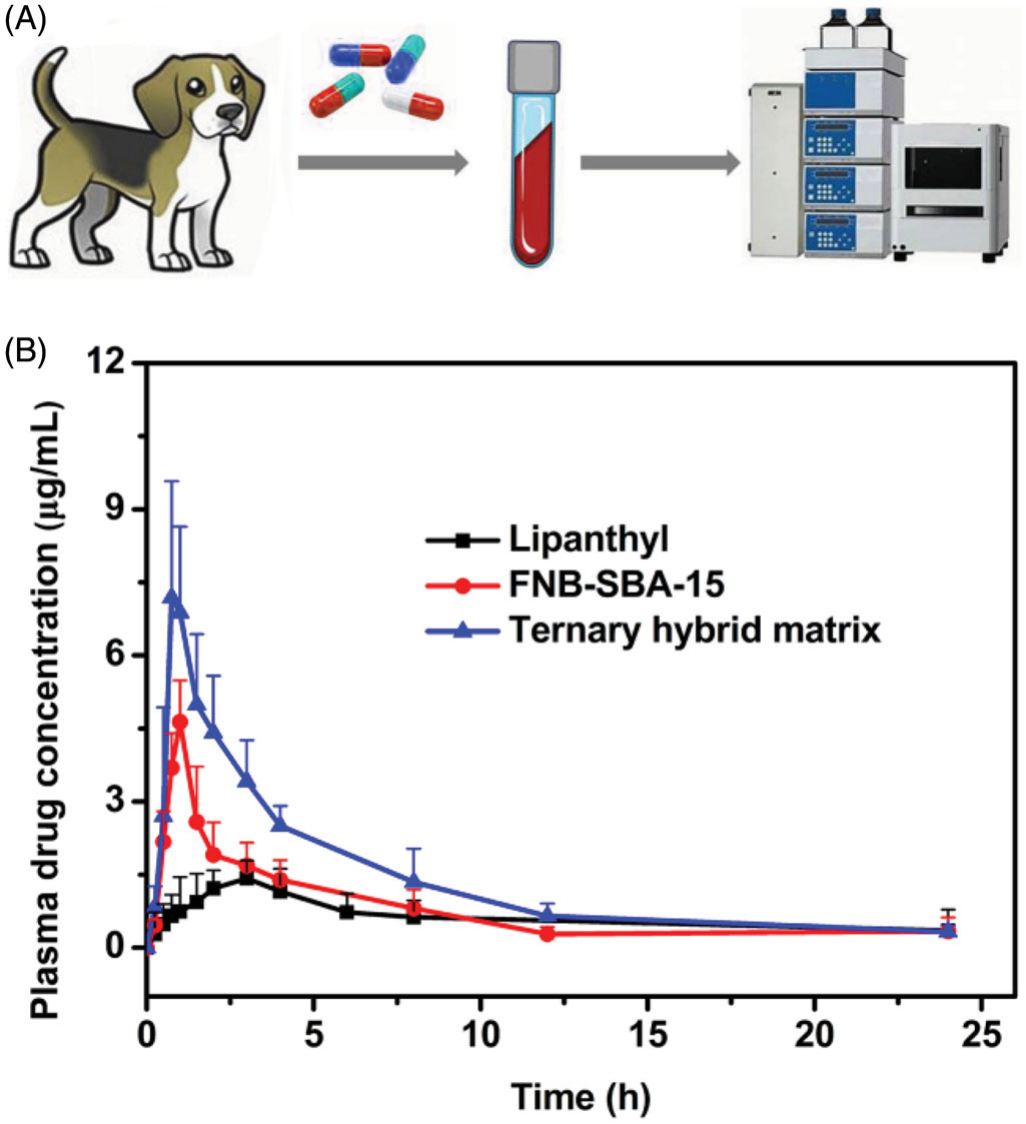
*In vivo* pharmacokinetic study. (A) Schematic diagram of pharmacokinetic study; (B) Plasma fenofibric acid concentration versus time following oral administration of the commercial capsule, FNB loaded SBA-15, and ternary hybrid matrix (mean ± S.D., *n* = 6).

The oral bioavailability of the ternary hybrid matrix, FNB-SBA-15, and the commercial capsule Lipanthyl was evaluated using six healthy beagle dogs. FNB is commonly metabolized into the main active metabolic fenofibric acid *in vivo* through the first pass effect. Therefore, the plasma concentration profiles of fenofibric acid versus time were calculated. As displayed in Figure 7(B), the plasma drug concentrations treated with the obtained solid dispersion formulations were remarkably greater than that of commercial micronized products. Moreover, the plasma drug concentrations of the hybrid matrix were higher than the FNB loaded SBA-15 without adding precipitation inhibitor at all time points, which is in good agreement with the *in vitro* dissolution test results.

The corresponding pharmacokinetic parameters were calculated and displayed in Table 3. The solid dispersion systems exhibited significantly higher C_max_ and AUC than commercial capsule (*p* < 0.05). Specifically, the C_max_ values of the FNB-SBA-15 and hybrid matrix were 2.93- and 4.75-times higher than that of the commercial FNB capsule, respectively. Meanwhile, the relevant AUC values were 1.23-and 2.36-times higher than that of the commercial product, respectively. Additionally, the hybrid matrix exhibited a significantly higher AUC value of 36.88 μg.h/mL in comparison with the FNB-SBA-15 sample without precipitation inhibitor (19.19 μg.h/mL).

**Table 3.** The calculated pharmacokinetic parameters after oral administration of commercial product, FNB-SBA-15, and ternary hybrid matrix (Mean ± S.D., *n* = 6).

PK parameter	Lipanthyl	FNB-SBA-15	Ternary hybrid matrix
*C*_max_ (μg/mL)	1.58 ± 0.43	4.64 ± 0.85	7.52 ± 1.84*
*T*_max_ (h)	2.25 ± 0.88	1.00 ± 0.00	1.05 ± 0.28
*t*_1/2_ (h)	12.40 ± 1.97	5.55 ± 3.40	7.47 ± 2.29
AUC_(0→∞)_ (μg.h/mL)	15.64 ± 3.34	19.19 ± 5.58	36.88 ± 3.84*

As is known to all, the oral bioavailability of water insoluble drugs is usually constrained by the slow dissolution rate. In this study, the successful conversion of crystalline FNB to its amorphous or molecular state in FNB-SBA-15 and hybrid matrix accelerated the dissolution rate, resulting in much higher concentration gradient between the GI tract and plasma, eventually leading to the enhanced oral absorption. For commercial micronized Lipanthyl^®^, although a relatively higher dissolution rate was obtained due to the size reduction as compared with raw FNB, the crystal characteristics was still retained with higher lattice energy, which leads to a lower bioavailability. Additionally, the organic-inorganic hybrid matrix could further increase the rate and extent of drug absorption, exhibiting even 90% higher relative bioavailability than that of FNB-SBA-15. The results were in consistent with the dissolution test, indicating that the hybrid matrix was able to maintain the supersaturated state in the GI tract for a longer time, which is quite beneficial for absorption. Both the *in vitro* and the *in vivo* results obviously indicated that the hybrid matrix using PVP VA64 as a supersaturation stabilizer is particularly effective in retarding FNB precipitation and ultimately enhancing drug absorption.

## 4. Conclusion

In this study, a novel supersaturated hybrid matrix containing water insoluble drug FNB was successfully constructed by combining inorganic mesoporous silica with polymeric precipitation inhibitor PVP VA 64. PVP VA 64 with similar hydrophobicity to the FNB was shown to effectively reduce the rate of nucleation and crystal growth and subsequently maintain the metastable supersaturated state. The results of pharmacokinetic study in beagle dogs showed that the hybrid matrix providing about a more than 90% increase in oral absorption. This study has indicated that the strategy of integrating mesoporous silica with appropriate polymeric addictive offers a modular platform to increase the absorption of water insoluble drugs.
